# Optimizations of Calcium‐Reinforced Nanophytosome of Pomegranate Extract Using Box–Behnken Design

**DOI:** 10.1002/fsn3.71229

**Published:** 2025-11-25

**Authors:** Ramesh Sedighi, Ghadir Rajabzadeh, Ali Rafe

**Affiliations:** ^1^ Department of Food Physics Research Institute of Food Science and Technology (RIFST) Mashhad Iran; ^2^ Department of Nanotechnology Research Institute of Food Science and Technology (RIFST) Mashhad Iran

**Keywords:** calcium ion enhancement, nanophytosomes, optimization, pomegranate, response surface methodology

## Abstract

This study developed reinforced nanophytosomes—innovative nanocarriers combining salts and lipids—using a thin layer hydration method to encapsulate bioactive compounds from pomegranate fruit extract (*Punica granatum L.*) from the red seed variety cultivated in Raver, Kerman, Iran. The extract was obtained via mechanical pressing and freeze‐drying, preserving its bioactive constituents. Laboratory analysis revealed high phenolic content (approximately 371 mg/g dry weight with ethanol and 370 mg/g with water), significant anthocyanins (around 300.68 mg/g), flavonoids (194.97 mg/100 g), and strong antioxidant activity (~91%), indicating its suitability for phytosomal formulation. To optimize nanophytosome properties, three key factors were studied: the ratio of extract to phosphatidylcholine (1:1, 1:2, 1:3), calcium chloride concentrations (0, 1.35 and 2.70 mM), and the extract/PC to ethanol ratio (0.4, 0.7, and 1.0 w/w). These parameters were evaluated using response surface method (RSM) with the Box–Behnken design in Design Expert software. The optimal formula was identified at a ratio of 1:3 pomegranate extract to PC, calcium chloride concentration of 2.70 mM, and ethanol ratio of 0.82 (w/w). The resulting nanophytosomes demonstrated a particle size of 127.67 nm, a zeta potential of −39.7 mV, mobility of −3.145 μm.cm/Vs, dispersity index of 0.357, and an encapsulation efficiency of 100%, achieving a desirability score of 0.860.

## Graphical abstract

Enhanced nanophytosome complexes are developed as protective nanocarriers for pomegranate fruit extract powder, ensuring the stability and preservation of bioactives. Optimal conditions yielded a 1:3 extract to phosphatidylcholine ratio, 2.70 mM CaCl_2_ concentration, and a solvent ratio of 0.82, resulting in nanophytosomes with a 127.67 nm particle size and 100% encapsulation efficiency. This novel approach enhances the stability and bioavailability of pomegranate bioactive compounds for potential applications in food and nutraceuticals.

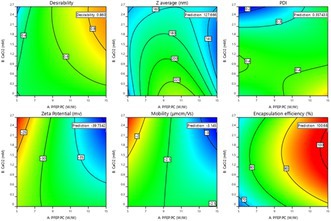

## 1 Introduction

Pomegranate (*Punica granatum* L.) is rich in bioactive compounds with significant nutritional and therapeutic properties, attracting extensive research interest (Kyriakoudi et al. 2021). However, their low stability, solubility, and bioavailability limit their applications in food, cosmetics, and pharmaceutics (Khan et al. 2013). Therefore, it is necessary to increase its stability through novel techniques.

To overcome these limitations, various nanostructures—including emulsions, liposomes, niosomes, and phytosomes—have been explored in drug delivery systems (Li et al. 2015). Among these, phytosomes stand out due to their superior stability and encapsulation efficiency (Yang et al. 2020). Phytosomes are stable complexes formed via electrostatic interactions (e.g., hydrogen bonding) between phospholipids (primarily phosphatidylcholine, PC) and plant polyphenols (Lua et al. 2019). Unlike liposomes, phytosomes chemically bind bioactive compounds, preventing leakage and enhancing targeted delivery (Amit et al. 2013; Ting et al. 2014; Nouri et al. 2020; Lu et al. 2025). Nanophytosomes have key advantages, such as improved bioavailability via systemic circulation (Bhosale et al. 2015), greater stability during storage and digestion, and controlled release in target tissues (Shivanand and Kinjal 2010; Giuliani et al. 2017; Kyriakoudi et al. 2021; Shen et al. 2024).

To load pomegranate extracts into phytosomes, a reaction medium is required. Traditional aprotic solvents (e.g., methylene chloride) lack hydrogen‐bonding capacity, limiting complex formation. However, modern formulations favor protic solvents such as ethanol, which improve safety and efficacy (Ghanbarzadeh et al. 2016; Shakeri and Sahebkar 2016).

Polyphenolic compounds of pomegranate extracts must be kept under suitable temperature conditions and have proper thermal stability (Amany et al. 2012). Freeze‐drying (lyophilization) is a preferred method for drying phytosomal complexes, despite its energy intensity. It preserves polyphenols, prevents oxidation, and extends shelf life (Antal et al. 2014; Mamdoh Ershidat et al. 2024).

Various phytosomal systems have been studied for medicinal and food applications, such as grape seed, blueberry, pine, and tea extracts (Yang et al. 2020), Raver Kerman pomegranate—an understudied Iranian cultivar—remains unexplored. The significance of the current work is introducing a stable, reinforced nanocarrier for pomegranate polyphenols, providing a consumer demand for natural, health‐promoting products (Carr et al. 2016), and expanding its applications in functional foods and nutraceutics. Therefore, the main aims of this research are to (i) characterize Raver Kerman pomegranate extract and (ii) and develop enhanced nanophytosomes to improve stability. The enhanced phytosomes will be able to be used in a variety of gel and drinking model systems, so that they can be used as food‐drug supplements for different sections of society. Accordingly, pomegranate extract, along with peels, was lyophilized, and then it was loaded into the enhanced phytosomal system through the thin‐layer hydration method. Finally, optimization was carried out by response surface methodology (Box‐Behnken design) using Design Expert software.

## 2 Materials and Methods

### 2.1 Materials

Ripped pomegranates ( *Punica granatum* , L.), red grain cultivar from Raver, Kerman, Iran, were provided by the local market. To prepare a nanophytosome system loaded with whole pomegranate fruit extract with peel and arils (PFE), various compounds including ethanol 96%, phosphatidylcholine (PC) (CAS Number: 97281‐47‐5), Folin–Ciocalteu, DPPH (2,2‐diphenyl‐1‐ picrylhydrazyl), di‐potassium hydrogen phosphate, sodium nitrite, citric acid, AlCl_3_, NaOH, NaCl, CaCl_2_, Na_2_CO_3_, quercetin, sodium acetate, deionized water, methanol, gallic acid were all provided by Merck (Darmstadt, Germany). All the other chemicals used for the analyses were of analytical grade.

### 2.2 Preparation of Pomegranate Fruit Extracts (PFE)

Pomegranate fruit extract was achieved with a double press machine (Rajasekar et al. 2012). The squeezing method was utilized to extract phenolic compounds from pomegranates to gently preserve bioactive compound integrity and minimize oxidation and cell damage. By squeezing, a high‐quality extract is created which maintains the natural flavor and composition of the fruit, catering to consumer preferences for minimally processed products and enhancing its suitability for functional food applications. The whole fruit extract was then filtered through a fabric filter. The samples were centrifuged (Universal 320R, Andreas Hettich GmbH & Co. KG, Germany) at 6500 rpm for 8 min, and the supernatant was collected. One portion of the extract was stored in a freezer at −25°C for further experiments. The other portion was lyophilized by a freeze dryer (Operon, FDU‐8606, Korea) for 72 h, and the powders were preserved at −18°C in Ziploc bags.

#### 2.2.1 Total Phenolic, Flavonoid and Anthocyanin Content of PFE

Total phenolic content (TPC) was assessed using a modified Folin–Ciocalteu colorimetric method (Tezcan et al. 2009). A 1000 ppm solution was obtained by dissolving 50 mg of pomegranate extract powder in 50 mL of deionized water in a volumetric flask. This solution was incubated in a water bath at 30°C for 1 h, and then centrifuged at 8500 rpm for 5 min, with the supernatant used for TPC analysis. The evaluation was performed against a gallic acid calibration curve spanning 0.003–0.1 mg/mL (Supplementary file). Samples were tested in triplicate, and results were expressed as mg gallic acid equivalent per gram of dry weight (mg/g GAE).

The total flavonoid content in plant extracts was determined using the AlCl_3_ colorimetric method (Tezcan et al. 2009). According to this method, 0.5 mL of diluted samples was mixed with 1.5 mL of methanol, and equal amounts of 10% AlCl_3_ and 1 M potassium acetate were added to the solution. The mixture was then diluted with 2 mL distilled water, vortexed, and left at room temperature for 30 min before measuring the absorbance at 415 nm. Quercetin was used to create a standard curve for determining the flavonoid content, and the results were expressed in mg/L and mg/g of dry matter (Data S1).

Total anthocyanin content was determined using a spectrophotometric method based on pH differences according to the previous work (Wrolstad et al. 2005). Initially, a stock solution of pomegranate extract powder was prepared. Following centrifugation for 10 min at 6500 rpm, 1 mL of the Falcon supernatant was extracted and mixed with KCl buffer of pH 1 (0.25 M KCl and concentrated HCl) to reach a total volume of 10 mL. Additionally, 1 mL of extract mixed with sodium acetate buffer with pH 4.5 (containing 0.4 M of sodium acetate and concentrated acetic acid) to a volume of 10 mL was incorporated. The absorption of these samples with buffer was measured using a spectrophotometer at 510 and 700 nm. Anthocyanidin levels were quantified in terms of cyanidin 3‐glucosidase, with the total anthocyanin content in the extract expressed in milligrams of cyanidin‐3‐glycoside per gram of dry powder according to the following Equations (1) and (2):

(1) $$∆A={\left(A_{510}-A_{700}\right)}_{\mathrm{at}\;\mathrm{pH}\;1}-{\left(A_{510}-A_{700}\right)}_{\mathrm{at}\;\mathrm{pH}\;4.5}$$

(2) $$C\left[\frac{\mathrm{mg}}{L}\right]=\frac{\left(∆A\times M_{w}\times D_{f}\times 1000\right)}{\left(\varepsilon \times L\right)}$$

where ∆A is the absorption difference, M_W_ is the molecular weight of the dominant anthocyanin (449.2), D_F_ is the dilution factor, ε is the molar absorption of the dominant anthocyanin, and L is the cell length in cm.

#### 2.2.2 Antioxidant Activity of PFE

The antioxidant capacity of pomegranate extract was evaluated using the DPPH (Tezcan et al. 2009) method with the free radical 2,2‐diphenyl‐1‐picrylhydrazyl (DPPH). A 0.008% DPPH solution was initially prepared for this analysis (Elfalleh et al. 2012). A stock solution of extract (1000 ppm) along with various solutions was prepared, which was protected from light by aluminum foil. In the control, 0.5 mL of methanol replaced the extract, while 0.5 mL of the extract was combined with 1.5 mL of DPPH solution. They were then vortexed and incubated in the dark for 30 min before measuring the absorbance. Calibration of the spectrophotometer was performed with 85% methanol (Data S1).

### 2.3 Nanophytosomes Preparation

Phytosomal nanoparticles, composed of phospholipids and pomegranate extract, were prepared using the thin layer hydration method, allowing precise control over their composition and size to achieve desired characteristics (Babazadeh et al. 2017; Dundar et al. 2023) Pomegranate fruit extract‐loaded nanophytosomes (PFE‐NP) were produced via the hydration‐sonication thin layer method as detailed in our previous work (Sedighi et al. 2025). Briefly, phospholipids were dissolved in ethanol with the extract, evaporated under vacuum to form a thin layer, then hydrated to yield a phytosome suspension.

In this study, reinforced nanophytosomes with pomegranate fruit extract (PFE‐NP) were prepared similarly. The extract‐to‐PC molar ratio (1:1, 1:2, 1:3) was optimized based on phenolic content. Mixtures were dissolved using a heated magnetic stirrer with 96% ethanol, while the extract powder was separately dissolved in foil‐wrapped deionized water to prevent light degradation. The extract was added dropwise to the phospholipid solution (800 rpm stirring), followed by calcium chloride. After 30 min stirring, the mixture was refrigerated (4°C, 24 h) for bond formation (Maryana et al. 2016). The solution was evaporated (45°C, vacuum) to a thin film, hydrated with water, and homogenized (ultra‐turrax, 10,000 rpm, 10 min). Sonication (7 mm probe, 50% amplitude, 10 min) further reduced particle size (Rasaie et al. 2014). Finally, the PFE‐NP complexes were lyophilized for 72 h and stored in a sealed Ziplock bag at −18°C.

### 2.4 Characterization of PFE‐NP

#### 2.4.1 Particle Size, Polydispersity, and ζ‐Potential

Particle size, polydispersity index (PDI), and ζ‐potential were analyzed using a dynamic light scattering (DLS) particle size analyzer (Nano ZS, ZEN3600, Malvern Instruments, UK). To minimize multiple scattering effects, samples were diluted 10‐fold with deionized water. Samples were then placed in a quartz cuvette and assessed at a 90^o^ scattering angle. Mean particle size was determined using Average‐*Z*, while nanoparticle uniformity and aggregation tendencies were evaluated using the PDI factor, which indicates overall particle consistency and behavior (Sze et al. 2003). ζ‐potential and electrophoretic mobility were also determined to check the surface charge and movement of charged particles of nano‐phytosomes (Barbosa et al. 2019).

#### 2.4.2 Encapsulation Efficiency

An indirect method was used to determine the encapsulation efficiency (EE) of pomegranate extract inside the nanophytosomes. For this purpose, appropriate concentrations of pomegranate extract were prepared with ethanol, and the absorption rate of the sample at the maximum absorption was read based on the data obtained from scanning by UV–vis spectrophotometer, and then the calibration curve was drawn, and its equation was determined (Babazadeh et al. 2017). The encapsulation efficiency, which is denoted as EE% of PFE when using PC as a carrier, was determined through the application of Dündar's method, albeit with some minor modifications to adapt the procedure to our specific requirements (Dundar et al. 2023). Initially, the total phenolic content (TPC) of pomegranate fruit extract was determined before and after the formation of nano‐phytosome. For this purpose, 1 g of PFE‐NP was mixed with 10 mL of ethanol/water (70/30 v/v) in a shaking water bath at 25°C for 2 h. Then the mixtures were centrifuged, and the supernatant was used for TPC analysis. EE was determined by Equation (3):

(3) $$\mathrm{EE}\left(\%\right)=\frac{{\mathrm{TPC}}_{\mathrm{PFE}}-{\mathrm{TPC}}_{\mathrm{NC}}}{{\mathrm{TPC}}_{\mathrm{PFE}}}\times 100$$

where TPC_PFE_ is the total phenolic content of pomegranate fruit extract, and TPC_NC_ is the total phenolic content of nano‐phytosome supernatant.

### 2.5 Experimental Design in Nanophytosome Optimization

A three‐level, three‐variable central composite design was employed to investigate the optimal conditions for encapsulating pomegranate fruit extract within a phytosomal nanostructure using the thin layer hydration method. This study aimed to determine the optimal ratio of pomegranate fruit extract to phospholipid, with ratios of 1:1, 1:2, and 1:3 (w/w), in combination with calcium chloride concentrations of 0, 1.35 and 2.70 mM, as well as varying ratios of pomegranate fruit extract and PC to ethanol solvent (0.4, 0.7, and 1 w/w). The response surface method (RSM) was applied using a Box–Behnken design, as detailed in Table 1. The independent variables included the pomegranate fruit extract to phospholipid ratio (A, w/w), calcium chloride concentration (B, mM), and the ratio of pomegranate fruit extract‐nanoparticles (PFE‐NP) to solvent (C, w/w). The dependent variables, which included particle size, zeta potential, and encapsulation efficiency, were analyzed using a quadratic model to assess the effects of the independent variables, and experimental data were fitted to the selected models. The statistical significance of the terms was evaluated using analysis of variance (ANOVA) for each response.

**TABLE 1:** Experimental design for PFE‐NP at three levels of PFE to PC ratio (A, w/w), calcium chloride concentration (B, mM), and the ratio of PFE‐NP to solvent (C, w/w).

Run	Variables			Responses				
	PEF: PC	CaCl_2_	PEF‐NP: solvent	*Z* average	PDI	ζ‐potential	Mobility	EE
	(w/w)	mM	(w/w)	(nm)	(−)	(mv)	(μmcm/Vs)	(%)
1	1:2	1.35	0.7	214.62	0.279	−34.6	−2.709	90.13
2	1:1	2.7	0.7	139.20	0.174	−21.86	−1.64	75.93
3	1:1	0	0.7	103.40	0.252	−28.16	−2.476	72.32
4	1:3	1.35	0.4	172.07	0.548	−31	−2.43	93.24
5	1:2	1.35	0.7	176.2	0.354	−33.2	−2.606	92.89
6	1:3	2.7	0.7	154.10	0.311	−40.1	−3.145	97.85
7	1:2	1.35	0.7	174.72	0.411	−33.9	−2.658	93.32
8	1:2	0	1	302.67	0.578	−29.6	−2.322	68.93
9	1:1	1.35	1	195.17	0.523	−24.7	−1.937	63.52
10	1:2	2.7	0.4	246.29	0.546	−30.4	−2.475	85.31
11	1:2	1.35	0.7	180.87	0.407	−30.3	−2.373	93.67
12	1:3	0	0.7	142.48	0.441	−31.6	−2.476	88.34
13	1:2	1.35	0.7	174.76	0.396	−30.2	−2.366	94.25
14	1:3	1.35	1	147.49	0.484	−37.4	−2.935	95.38
15	1:1	1.35	0.4	176.10	0.522	−23.7	−1.859	73.91
16	1:2	0	0.4	199.11	0.422	−27.3	−2.143	72.65
17	1:2	2.7	1	176.2	0.279	−30.7	−2.406	75.00

To optimize multiple responses, desirability functions were employed in the following manner: (i) experiments were performed and response models (y_i_) for all m responses were fitted; (ii) individual desirability functions (d_i_) for each response were defined; and (iii) the overall desirability was maximized concerning the controllable factors. The general approach involved converting each response, yi, into an individual desirability function, d_i_, which ranges from 0 to 1, where d_i_ equals 1 at the target value and drops to 0 outside an acceptable region. The design variables were selected to maximize overall desirability (D_o_) as follows: Do = (d_1_ d_2_ … d_m_) ^(1/m), where m represents the number of responses and D_o_ indicates overall desirability. Since the individual desirability functions are not differentiable, overall desirability was computed using Design‐Expert for evaluating optimal processing conditions (Borror et al. 2002).

### 2.6 Statistical Analysis

The collected data in Table 1 were processed using the commercial statistical package Design Expert software Version 12 (Stat‐Ease Inc., Minneapolis, MN, USA). This software facilitated analysis of variance (ANOVA), mathematical modeling, regression analysis, and optimization. Response surface plots were generated to illustrate different interactions. The contour plots for all responses were superimposed, allowing us to identify the regions that best meet all constraints and thereby determine the optimum conditions. The data were averaged and presented as mean ± standard deviation (SD). To assess significant differences between the means of each treatment, Duncan's multiple range test was applied at the 5% significance level.

## 3 Results and Discussion

### 3.1 Total Phenolic Content of PFE

The whole pomegranate fruit extract (PFE), including both peels and arils, demonstrated a total phenolic content (TPC) of 371.19 ± 2.28 mg/g when extracted with ethanol, and 370.04 ± 3.50 mg/g when extracted with deionized water. Recent analyses of TPC across different parts of the pomegranate have revealed distinct values for its components. For instance, it has been reported that pomegranate peel contains approximately 58.63 ± 0.129 mg/g GAE (Farag et al. 2014; Bacha et al. 2024). The seeds are estimated to possess around 101 mg/g GAE (Kumar et al. 2021). Similar results have also been reported for Kandhari pomegranate peel (289.40 mg/g GAE), which was significantly higher than that of the Badana variety (255.35 mg/g GAE) (Khalil et al. 2017). However, other studies highlighted even higher levels; for example, TPC levels in Iranian pomegranate peel extracts have been reported as 66.38–181.41 mg/g GAE among different genotypes, notably genotype IPP23, which exhibited a high phenolic content (161.5 mg/g GAE) (Tavakoli et al. 2024). These differences in phenolic content from numerous researchers can be attributed to the extraction method employed. Furthermore, differences in the phenolic content among various extracts can be attributed to their ability to disrupt the bonds between tannin molecules and proteins in plant tissues, as well as to the rapid dissolution of hydrolysable tannins (Glen et al. 2016). In a study, TPC of pomegranate peel extracts was evaluated using different solvents and the aqueous extract showed a phenolic content of approximately 200 mg/g GAE. The extracts obtained with PC: pomegranate peel at ratios of 1:1 and 1:2 had phenolic contents of around 250 mg/g GAE, while the PC: glycerol at ratio 1:2 extract exhibited the highest phenolic content, approximately 500 mg/g GAE (de Oliveira et al. 2024).

Comparative analyses with other fruits have shown varying levels of total phenolic compounds; for instance, the total phenolic content in blueberries ranges from approximately 0.597 mg/g to 1.417 mg/g, which is comparable to or higher than that of pomegranate, depending on cultivar (Zhang et al. 2016). This showcases that pomegranates can have high phenolic content compared to other fruits, where average values hover around 50 mg/g GAE or less for typical fruits like apples and oranges (Elfalleh et al. 2012).

### 3.2 Antioxidant Activity of PFE

Regarding antioxidant activity, pomegranate peel extracts tend to outperform many other fruits when evaluated through the DPPH assay. It has been noted that DPPH EC50 values range from 4.1 to 14.4 μg/ml across different genotypes (Tavakoli et al. 2024). In another study, Faraj et al. reported antioxidant activity using the DPPH method, with EC50 values of 20.296 ± 0.005 μg/mL for pomegranate peel juice and 3.081 ± 0.009 μg/mL for pomegranate leaf juice (Farag et al. 2014). In contrast, it has been found that pomegranate extracts displayed antioxidant inhibition as high as 78.23% in the Kandhari peel at lower concentrations (Khalil et al. 2017). Other fruits, such as apples and bananas, typically exhibit much higher EC50 values (around 20–25 μg/mL), indicating lower antioxidant activity (Elfalleh et al. 2012). Our current research indicates that the total antioxidant activity of the whole pomegranate fruit is 90.98% ± 0.80%.

### 3.3 Anthocyanin and Flavonoid Content of PFE

Total anthocyanin content of PFE was 300.68 ± 7.56 mg/g. Although pomegranate peels also exhibit significant anthocyanin content, identifying concentrations up to 102.2 mg CGE/g in various pomegranate samples (Tavakoli et al. 2024). This is in contrast to many other fruits, such as apples, which generally contain lower anthocyanin levels (usually around 5–10 mg CGE/g) (Elfalleh et al. 2012). Blueberry anthocyanin levels are generally between 0.138 and 0.493 mg/g of dry weight, often exceeding those in pomegranate, highlighting the deep coloration and antioxidant capacity of blueberries (Zhang et al. 2016).

The flavonoid content of PFE whole pomegranate fruit (peels and arils) was 194.97 ± 5.23 mg/100 g, expressed as quercetin equivalents for this study. In contrast, pomegranate peel juice typically shows 47.32 ± 0.032 mg/g dry weight (Farag et al. 2014). Regarding total flavonoid content (TFC), the highest content was recorded in pomegranate peel extracts from the Kandhari variety at 58.63 mg/g RE (Khalil et al. 2017). TFC levels have also been found ranging 38.5–144.13 mg/g RE, with genotype IPP_23_ having a TFC of 131.6 mg/g RE (Tavakoli et al. 2024). In a study, the flavonoid content of barberry was reported to be between 400 and 900 mg/100g, expressed as quercetin equivalents (Sadeghi 2020), The flavonoid content in blueberry juice is approximately 5.14–27.31 mg/100g, expressed as quercetin equivalents (Zhang et al. 2016). Examining the results of several studies revealed that the total flavonoid content, much like total phenol content, varies depending on the cultivar and the specific part of the plant being extracted. In all cultivars studied, the highest levels of total flavonoids are associated with the skin extract. This variability is largely influenced by heredity and the genetic potential of the variety, making it a key characteristic of different cultivars.

### 3.4 Optimizing PFE‐NP Treatments Using Box–Behnken

This study utilized Design‐Expert version12 to optimize pomegranate extract encapsulation in a phytosomal nanostructure via Box–Behnken design (see Table 1). The software facilitated efficient experimental planning, interaction analysis, and precise response surface modeling, streamlining the process, reducing resource consumption, and improving optimization accuracy in nanotech formulation. ANOVA results for particle size, size distribution, zeta potential, mobility, and encapsulation efficiency are detailed in Tables 2 and 5, 6, 7, 8, respectively. Statistical values of different variables on responses including particle size, ζ‐potential, electrophoretic mobility, and encapsulation efficiency are given in Table 3. The optimized conditions predicted by the software are summarized in Table 4. The optimized conditions predicted by the software are summarized in Section 3.6. These researchers used the Design of Experiment approach with specialized software to optimize nanophytosomal formulations, improving characteristics such as particle size, drug loading capacity, and stability. Key process parameters like addition rate, temperature, and phospholipid concentration significantly influenced the final properties. This methodology led to the development of effective and stable drug delivery systems with enhanced bioavailability (Shariare et al. 2020; Metkari et al. 2023).

**TABLE 2:** ANOVA for reduced cubic model, response *Z* average.

Source	Sum of squares	df	Mean square	*F*‐value	*p*	
Model	30683.47	11	2789.41	10.18	0.0095	Significant
A‐PFEP: LC	668.48	1	668.48	2.44	0.1790	
B‐CaCL2	1571.73	1	1571.73	5.74	0.0620	
C‐PFEP+LC: SOLVENT	97.72	1	97.72	0.3568	0.5763	
AB	146.17	1	146.17	0.5337	0.4978	
AC	476.33	1	476.33	1.74	0.2444	
BC	7538.58	1	7538.58	27.53	0.0033	
A^2^	12232.24	1	12232.24	44.66	0.0011	
B^2^	83.77	1	83.77	0.3059	0.6040	
C^2^	7559.88	1	7559.88	27.60	0.0033	
A^2^B	2006.93	1	2006.93	7.33	0.0424	
AB^2^	1396.30	1	1396.30	5.10	0.0735	
Residual	1369.37	5	273.87			
Lack of fit	189.93	1	189.93	0.6441	0.4672	Not significant
Pure error	1179.44	4	294.86			
Cor total	32052.85	16				

**TABLE 3:** Statistical values of different variables on responses including particle size, ζ‐potential, electrophoretic mobility, and encapsulation efficiency.

	*Z* average, nm	PDI (−)	ζ‐potential	Mobility	EE, %
Mean ± SD	180.91 ± 16.55	0.41 ± 0.04	−30.51 ± 1.72	−2.41 ± 0.16	83.92 ± 2.40
*R* ^2^	0.96	0.95	0.94	0.92	0.98
Adjusted *R* ^2^	0.86	0.86	0.86	0.82	0.95
Predicted *R* ^2^	NA	0.77	0.75	0.41	0.75
Adeq precision^a^	13.98	11.60	13.51	12.51	19.42
CV %	9.15	10.75	5.64	6.58	2.86

^a^ Adeq Precision measures the signal‐to‐noise ratio. A ratio greater than 4 is desirable. Your ratio of 13.981 indicates an adequate signal. This model can be used to navigate the design space.

**TABLE 4:** Comparison of optimal values obtained by RSM and experiments.

Condition	PEF: PC ratio (w/w)	CaCL_2_ mM	PEF‐PC: solvent (w/w)	Particle size (nm)	PDI (−)	Zeta potential (mV)	Mobility μmcm/Vs	EE, %	Desirability
Optimal	1:3	2.269	0.82	127.67	0.357	−39.7	−3.145	100.680	0.860
Experimental	1:3	2.70	0.82	128.90	0.410	−40.1	−3.145	97.85	

In one study, nanophytosome optimization was performed with a Box–Behnken design (3 factors, 3 levels). Independent variables: phospholipid amblypherone ratio (X_1_), temperature (X_2_), time (X_3_). Dependent variables: complex formation rate (Y_1_), partition coefficient (Y_2_) (Table 1). Fifteen batches were prepared and evaluated. Data were statistically analyzed and validated with Design Expert and optimal parameters were determined with utility functions and response surface plots. A nonlinear quadratic model equation was created (Ittadwar and Puranik 2017).

In another study, a gefitinib‐phospholipid complex was produced using the solvent evaporation method and the parameters were optimized using Box–Behnken design (BBD) software and the formulation was subjected to in vitro studies. The results showed that the globule size and zeta potential for the optimized batch were 165.6 nm and −24.4 mV, respectively. SEM morphology showed spherical nanoparticles (Mohit Kumar et al. 2023).

#### 3.4.1 Particle Size of Reinforced PFE‐NP

The size of lipid vesicles is influenced by several factors, including the ratio of bioactive compounds to lipids, environmental conditions (temperature, pressure, time, and sonication cycles), production methods, and formulation conditions (pH, temperature, ionic strength, and buffer compounds). These factors affect particle size and shape, impacting their aggregation, colloidal spreading, adherence, and emulsion stability (Molaveisi et al. 2020).

Pomegranate fruit extract contains charged groups capable of forming hydrogen or ionic bonds with the polar head of PC. The hydrophobic portion of the active compounds can reside within the phospholipid monolayer, interacting with its hydrocarbon chains. This interaction significantly influences the size of phytosome particles (Rafiee et al. 2017). This study investigated three key factors affecting the size of nanophytosomes: Factor A (w/w PFE: PC ratio), Factor B (CaCl_2_ concentration in mM), and Factor C (PFE‐NP: solvent ratio). The analysis in the context of calcium ion–enhanced nanoliposome formulations reveals significant interactions that affect the average nanoparticle size (*Z*‐average) Equation (4).

(4) $$\begin{array}{c} Z-\text{average}=-102.55+77.46\cdot \mathrm{PFE}:\mathrm{PC}+234.85\cdot {\text{CaCl}}_{2}\\ -430.03\cdot \mathrm{PFE}-\mathrm{NP}:\text{solvent}-27.50\cdot \mathrm{PFE}:\mathrm{PC}\cdot {\text{CaCl}}_{2}\\ -7.28\cdot \mathrm{PFE}:\mathrm{PC}\cdot \mathrm{PFE}-\mathrm{NP}:\text{Solvent}-107.19\cdot {\text{CaCl}}_{2}\cdot \mathrm{PFE}\\ -\mathrm{NP}:\text{solvent}-3.42\cdot \mathrm{PFE}:{\mathrm{PC}}^{2}-26.55\cdot {{\text{CaCl}}_{2}}^{2}+470.81\cdot \mathrm{PFE}\\ -\mathrm{NP}:{\text{Solvent}}^{2}+0.94\cdot \mathrm{PFE}:{\mathrm{PC}}^{2}\cdot \text{CaCl2}+2.90\cdot \mathrm{PFE}:\mathrm{PC}\cdot {\text{CaCl2}}^{2} \end{array}$$

**FIGURE 1:**
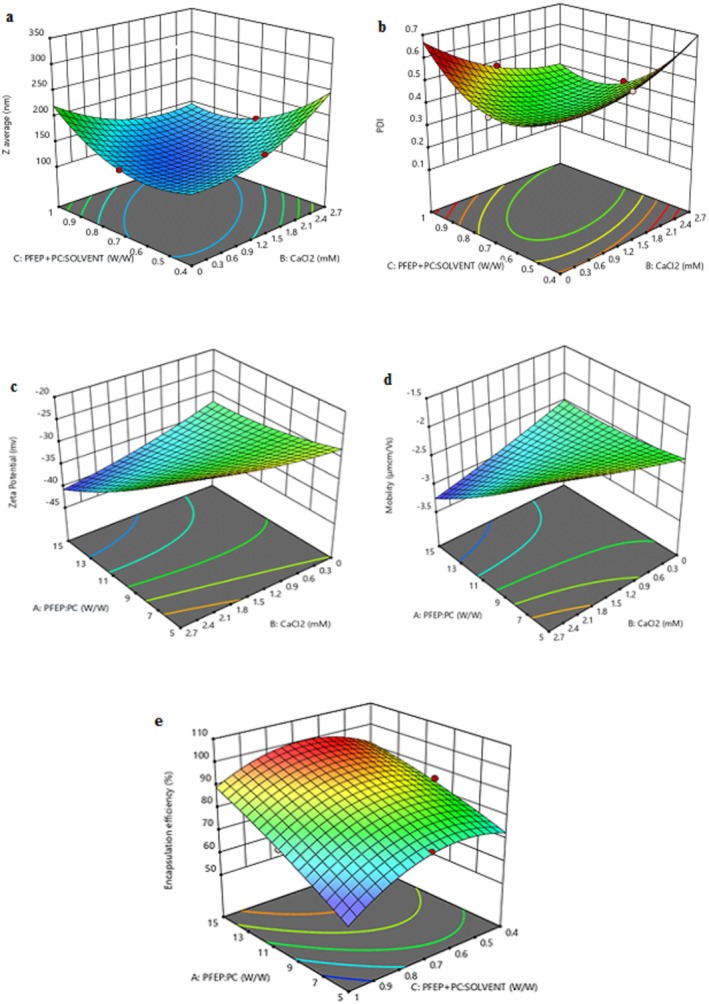
3D plot of particle size (a), PDI (b), ζ‐potential (c), mobility (d), and encapsulation efficiency (e) of nanophytosomes loaded with PFE using various PFE: PC ratios (a), CaCl_2_ (b), and PFE‐NP: Solvent ratio (c).

According to Equation (4) and Table 2, the interaction between B and C (*F*‐value = 27.53, *p*‐value = 0.003), as well as the squared terms A^2^ and C^2^ (*F*‐values of 44.66 and 27.60, respectively, with *p*‐values of 0.001), indicates that these factors play critical roles in determining particle dimensions. The significant positive coefficient associated with CaCl_2_ suggests that increasing its concentration tends to enlarge the nanoparticle size, likely due to enhanced stability or aggregation effects. Conversely, the negative coefficients related to the PFE‐NP: solvent imply a complex relationship that could favor smaller sizes under optimal conditions Figure 1a. The findings align with previous studies on nanoparticle formulations, which emphasize the critical role of ionic strength and solvent composition in controlling particle dimensions. These insights were drawn from the analysis of 17 treatments using Design Expert software (Table 1), emphasizing the importance of understanding these interactions for developing effective nanocarrier systems (Molaveisi et al. 2020).

Previous studies, including those by Mohit, Kumar, and colleagues in 2023, have highlighted the significance of particle size in nanocarrier systems. Specifically, the gefitinib‐phospholipid complex with a particle size of approximately 165.6 nm demonstrated enhanced sustained release (Mohit Kumar et al. 2023), thereby improving oral bioavailability. Similarly, Telange et al. (2017) reported that apigenin‐phospholipid phytosomes with a particle size of around 107.08 nm are optimal for oral drug delivery. Based on these findings and previous literature, the particle size of the pomegranate fruit extract‐containing nanophytosomes produced in this study is also within a suitable range for effective oral administration (Telange et al. 2017).

#### 3.4.2 Polydispersity Index of Reinforced PFE‐NP

Particle size distribution is one of the important indicators to determine the stability of nanocarriers (Cheng 2021). Values above 0.5 indicate a heterogeneous system, and values less than 0.3 indicate a homogeneous system, which indicates that the produced system is homogeneous (Rafiee et al. 2017). The analysis of the factors A (PFE: PC), B (CaCl_2_), and C (PFE‐NP: solvent) on the polydispersity index (PDI) demonstrates several significant interactions affecting the uniformity of the nanoparticle size distribution Equation (5).

(5) $$\begin{array}{c} \mathrm{PDI}=0.76+0.01\cdot \mathrm{PFE}:\mathrm{PC}+0.54\cdot {\text{CaCl}}_{2}-1.97\cdot \mathrm{PFE}+\mathrm{PC}:\text{Solvent}\\ -0.03\cdot \mathrm{PFE}:\mathrm{PC}\cdot {\text{CaCl}}_{2}-0.01\cdot \mathrm{PFE}:\mathrm{PC}\cdot \mathrm{PFE}-\mathrm{NP}:\text{Solvent}\\ -0.26\cdot {\text{CaCl}}_{2}\cdot \mathrm{PFE}-\mathrm{NP}:\text{Solvent}+0.0005\cdot \mathrm{PFE}:{\mathrm{PC}}^{2}\\ -0.15\cdot {{\text{CaCl}}_{2}}^{2}+1.68\cdot \mathrm{PFE}-\mathrm{NP}:{\text{Solvent}}^{2}\\ +0.01\cdot \mathrm{PFE}:\mathrm{PC}\cdot {{\text{CaCl}}_{2}}^{2} \end{array}$$

The model shows a significant *F*‐value of 10.92 (*p*‐value = 0.0042), indicating a strong fit for predicting PDI based on the selected factors Table 5. Notably, the interaction between B and C (*F*‐value = 23.19, *p*‐value = 0.003) suggests that the combination of calcium chloride concentration and solvent ratio plays a crucial role in reducing the PDI, promoting a more homogeneous particle size distribution. Additionally, the squared term for C (*F*‐value = 49.97, *p*‐value = 0.0004) indicates that higher levels of PFE‐NP: solvent are particularly effective in decreasing PDI, suggesting a favorable impact on the stability of the formulation. The insignificance of other main effects, such as A and C, implies that these should be considered secondary to the significant interactions Figure 1b. Overall, understanding these factors is essential for optimizing the formulation of calcium‐enhanced nanocarriers, as confirmed by findings in related literature.

**TABLE 5:** ANOVA for reduced cubic model, response PDI.

Source	Sum of squares	df	Mean square	*F*‐value	*p*	
Model	0.2106	10	0.0211	10.92	0.0042	Significant
A‐PFEP: PC	0.0000	1	0.0000	0.0219	0.8872	
B‐CaCl2	0.0100	1	0.0100	5.19	0.0630	
C‐PFEP+PC: SOLVENT	0.0038	1	0.0038	1.96	0.2109	
AB	0.0006	1	0.0006	0.2986	0.6045	
AC	0.0011	1	0.0011	0.5475	0.4873	
BC	0.0447	1	0.0447	23.19	0.0030	
A^2^	0.0009	1	0.0009	0.4621	0.5220	
B^2^	0.0099	1	0.0099	5.12	0.0642	
C^2^	0.0964	1	0.0964	49.97	0.0004	
AB^2^	0.0241	1	0.0241	12.49	0.0123	
Residual	0.0116	6	0.0019			
Lack of fit	0.0008	2	0.0004	0.1583	0.8587	Not significant
Pure error	0.0107	4	0.0027			
Cor total	0.2222	16				

#### 3.4.3 Zeta Potential of Reinforced PFE‐NP

The analysis of the factors A (PFE: PC), B (CaCl_2_), and C (PFE‐NP: solvent) on the ζ‐potential reveals critical insights into the stability of the nanoparticle formulations Equation (6).

(6) $$\begin{array}{c} \zeta -\text{potential}=-14.01-0.60\cdot \mathrm{PFE}:\mathrm{PC}+2.76\cdot {\text{CaCl}}_{2}\\ -29.27\cdot \mathrm{PFE}-\mathrm{NP}:\text{Solvent}-0.55\cdot \mathrm{PFE}:\mathrm{PC}\cdot {\text{CaCl}}_{2}\\ -0.90\cdot \mathrm{PFE}:\mathrm{PC}\cdot \mathrm{PFE}-\mathrm{NP}:\text{Solvent}+1.23\cdot {\text{CaCl}}_{2}\cdot \mathrm{PFE}\\ -\mathrm{NP}:\text{Solvent}+0.05\cdot \mathrm{PFE}:{\mathrm{PC}}^{2}+0.47\cdot {{\text{CaCl}}_{2}}^{2}\\ +23.17\cdot \mathrm{PFE}-\mathrm{NP}:{\text{Solvent}}^{2} \end{array}$$

The model demonstrates a significant *F*‐value of 12.30 (*p*‐value = 0.0016), indicating a robust predictive capability Table 6. Notably, the main effect of A (*F*‐value = 73.42, *p*‐value < 0.0001) is highly significant, showing that variations in PFE: PC concentration substantially influence the ζ‐potential, suggesting that higher ratios lead to more negative ζ‐potential values, which could enhance stability through electrostatic repulsion. The interaction between A and B (*F*‐value = 18.51, *p*‐value = 0.0036) also plays a significant role, indicating that the combination of PFE: PC and CaCl_2_ critically affects the ζ‐potential, possibly due to the ionic strength modifying the surface charge dynamics. Moreover, the squared term of C (*F*‐value = 6.19, *p*‐value = 0.0417) underscores the importance of PFE‐NP: solvent levels in optimizing stability, suggesting that higher solvent ratios may lead to more favorable ζ‐potential outcomes. These findings emphasize the importance of optimizing these factors in developing stable nanocarrier systems, corroborating previous research on the effects of formulation components on nano‐stability (Figure 1c).

**TABLE 6:** ANOVA for quadratic model, response zeta potential.

Source	Sum of squares	df	Mean square	*F*‐value	*p*	
Model	327.42	9	36.38	12.30	0.0016	significant
A‐PFEP: LC	217.15	1	217.15	73.42	< 0.0001	
B‐CaCL2	5.12	1	5.12	1.73	0.2297	
C‐PFEP+LC: SOLVENT	12.50	1	12.50	4.23	0.0789	
AB	54.76	1	54.76	18.51	0.0036	
AC	7.29	1	7.29	2.46	0.1604	
BC	1.0000	1	1.0000	0.3381	0.5792	
A^2^	5.62	1	5.62	1.90	0.2106	
B^2^	3.08	1	3.08	1.04	0.3416	
C^2^	18.30	1	18.30	6.19	0.0417	
Residual	20.70	7	2.96			
Lack of fit	3.73	3	1.24	0.2933	0.8293	Not significant
Pure error	16.97	4	4.24			
Cor total	348.12	16				

It has been reported a zeta potential of −28.53 mV for chlorogenic acid‐phospholipid complexes (Bothiraja et al. 2014), and similarly a zeta potential of −22.35 mV for apigenin‐phospholipid complexes has been observed, indicating good physical stability. These studies collectively suggest that the nanophytosomes in this research, with comparable zeta potential values, possess favorable stability characteristics for oral delivery (Telange et al. 2017).

#### 3.4.4 Mobility of Reinforced PFE‐NP

The analysis of factors A and B on the mobility of nanoparticles reveals significant insights Equation (7).

(7) $$\begin{array}{c} \text{Mobility}=-1.35-0.01\cdot \mathrm{PFE}:\mathrm{PC}+0.38\cdot {\text{CaCl}}_{2}-2.51\cdot \mathrm{PFE}\\ -\mathrm{NP}:\text{Solvent}-0.06\cdot \mathrm{PFE}:\mathrm{PC}\cdot {\text{CaCl}}_{2}-0.07\cdot \mathrm{PFE}:\mathrm{PC}\cdot \mathrm{PFE}\\ -\mathrm{NP}:\text{Solvent}+0.003\cdot \mathrm{PFE}:{\mathrm{PC}}^{2}+0.02\cdot {{\text{CaCl}}_{2}}^{2}+1.94\cdot \mathrm{PFE}\\ -\mathrm{NP}:{\text{Solvent}}^{2} \end{array}$$

The model demonstrates a significant *F*‐value of 9.04 (*p*‐value = 0.0042), indicating a robust predictive capability Table 7. The main effect of factor A is highly significant (*F*‐value = 46.99, *p*‐value = 0.0002), suggesting that variations in the PFE: PC ratio profoundly affect the mobility of the nanoparticles, with higher ratios leading to decreased mobility. Additionally, the interaction between A and B (*F*‐value = 22.53, *p*‐value = 0.0021) highlights that the combined effects of PFE: PC and CaCl_2_ significantly influence mobility, suggesting that optimal formulations may require careful balancing of these factors. The relationship between mobility and the PFEP + PC: solvent term (with a squared term significant at *F*‐value = 5.13, *p*‐value = 0.0580) also points to an important consideration in formulation, indicating the potential for increased stability at specific solvent ratios. These findings align with existing literature, which indicates the critical role of formulation parameters in modulating the behavior of nanoparticles (Figure 1d).

**TABLE 7:** ANOVA for quadratic model, response mobility.

Source	Sum of squares	df	Mean square	*F*‐value	*p*	
Model	2.05	9	0.2272	9.04	0.0042	Significant
A‐PFEP: LC	1.18	1	1.18	46.99	0.0002	
B‐CaCL2	0.0078	1	0.0078	0.3083	0.5960	
C‐PFEP+LC: SOLVENT	0.0600	1	0.0600	2.39	0.1662	
AB	0.5663	1	0.5663	22.53	0.0021	
AC	0.0456	1	0.0456	1.81	0.2201	
BC	0.0154	1	0.0154	0.6117	0.4598	
A^2^	0.0251	1	0.0251	0.9984	0.3510	
B^2^	0.0040	1	0.0040	0.1605	0.7007	
C^2^	0.1289	1	0.1289	5.13	0.0580	
Residual	0.1759	7	0.0251			
Lack of fit	0.0710	3	0.0237	0.9013	0.5146	Not significant
Pure error	0.1050	4	0.0262			
Cor total	2.22	16				

#### 3.4.5 Encapsulation Efficiency of Reinforced PFE‐NP

The analysis of encapsulation efficiency based on factors A (PFE: PC), B (CaCl_2_), and C (PFE‐NP: solvent) highlights critical considerations for optimizing nanophytosome formulations Equation (8).

(8) $$\begin{array}{c} \text{Encapsulation Efficiency}=19.75+1.75\cdot \mathrm{PFE}:\mathrm{PC}\\ +14.93\cdot {\text{CaCl}}_{2}+126.81\cdot \mathrm{PFE}-\mathrm{NP}:\text{Solvent}\\ +0.22\cdot \mathrm{PFE}:\mathrm{PC}\cdot {\text{CaCl}}_{2}+2.09\cdot \mathrm{PFE}:\mathrm{PC}\cdot \mathrm{PFE}\\ -\mathrm{NP}:\text{Solvent}-4.07\cdot {\text{CaCl}}_{2\cdot }\mathrm{PFE}-\mathrm{NP}:\text{Solvent}\\ -0.06\cdot \mathrm{PFE}:{\mathrm{PC}}^{2}-4.19\cdot {{\text{CaCl}}_{2}}^{2}-108.21\cdot \mathrm{PFE}\\ -\mathrm{NP}:{\text{Solvent}}^{2} \end{array}$$

The model demonstrates a significant *F*‐value of 37.60 (*p*‐value < 0.0001), indicating that these factors notably influence encapsulation efficiency Table 8. Factor A has a strong positive effect (*F*‐value = 172.45, *p*‐value < 0.0001), suggesting that increasing the PFE: PC ratio enhances drug loading capacity. Factor B also significantly contributes (*F*‐value = 22.02, *p*‐value = 0.0022), with higher CaCl_2_ concentrations improving nanoparticle stability and encapsulation. Factor C is impactful (*F*‐value = 10.78, *p*‐value = 0.0134), emphasizing the role of solvent composition in drug incorporation. The interaction between A and C (*F*‐value = 6.82, *p*‐value = 0.0349) indicates that their combined effects are crucial for optimization. The squared term for C shows that higher solvent ratios can enhance efficiency (*F*‐value = 69.35, *p*‐value < 0.0001), necessitating careful solvent adjustments. Regression analysis provides valuable insights into model prediction, with significant coefficients for factors A (+1.752) and B (+14.9) reflecting their positive impacts. The coefficient for C's squared term (−108.20556) indicates a non‐linear relationship, where too much solvent may reduce efficiency. The model's *R* ^2^ value of 0.979 suggests it explains around 98% of the variability, while the predicted *R* ^2^ of 0.7502 indicates some areas for improvement Table 3. These findings emphasize the importance of balancing these factors to maximize encapsulation efficiency in nanocarrier systems and align with existing literature on polymer‐based formulations (Figure 1e).

**TABLE 8:** ANOVA for quadratic model, response encapsulation efficiency.

Source	Sum of squares	df	Mean square	*F*‐value	*p*	
Model	1948.59	9	216.51	37.60	< 0.0001	Significant
A‐PFEP: PC	993.02	1	993.02	172.45	< 0.0001	
B‐CaCl2	126.80	1	126.80	22.02	0.0022	
C‐PFEP+PC: SOLVENT	62.05	1	62.05	10.78	0.0134	
AB	8.70	1	8.70	1.51	0.2587	
AC	39.25	1	39.25	6.82	0.0349	
BC	10.86	1	10.86	1.89	0.2121	
A^2^	10.79	1	10.79	1.87	0.2133	
B^2^	245.83	1	245.83	42.69	0.0003	
C^2^	399.32	1	399.32	69.35	< 0.0001	
Residual	40.31	7	5.76			
Lack of fit	30.06	3	10.02	3.91	0.1105	Not significant
Pure error	10.25	4	2.56			
Cor total	1988.90	16				

The encapsulation efficiency of micronutrients is primarily influenced by their solubility within the lipid matrix, with higher solubility correlating with increased encapsulation percentages. Nanophytosomes' lipid‐based structure enhances the solubility of bioactive compounds, thereby improving encapsulation efficiency. In a study, the nanophytosomes possess a crystalline (solid lipid) structure reinforced by ionic calcium bonds between lipid layers. Calcium ion complexation further stabilizes the nanocarriers and facilitates the encapsulation of diverse bioactive agents (Sedighi et al. 2025).

Previous research demonstrates the efficacy of calcium‐enhanced nanophytosomes for encapsulating various compounds: for instance, thyme essential oil components thymol and carvacrol achieved encapsulation efficiencies of approximately 46.3% and 50.9%, respectively (Asprea et al. 2017) Khair et al. (2020) reported an efficiency of 84.78% in insulin‐loaded nanocapsules formulated with soy lecithin and cholesterol using the thin‐film hydration method (Khair et al. 2020). Similarly, amphotericin B nanocapsules exhibited about 55% efficiency, aligning well with the current findings. Overall, the increased encapsulation capacity of calcium‐enhanced nanophytosomes is attributed to their lipid architecture, which improves the solubility and loading of bioactive compounds such as those derived from pomegranate, fruits, plant extracts, and vitamins. The ionic bonds formed by calcium between lipid layers contribute to the structural stability and suitability of these nanocarriers for delivering various bioactives in pharmaceutical and nutraceutical applications.

### 3.5 Analysis of Contour Plots for Desired Reinforced PFE‐NP

**FIGURE 2:**
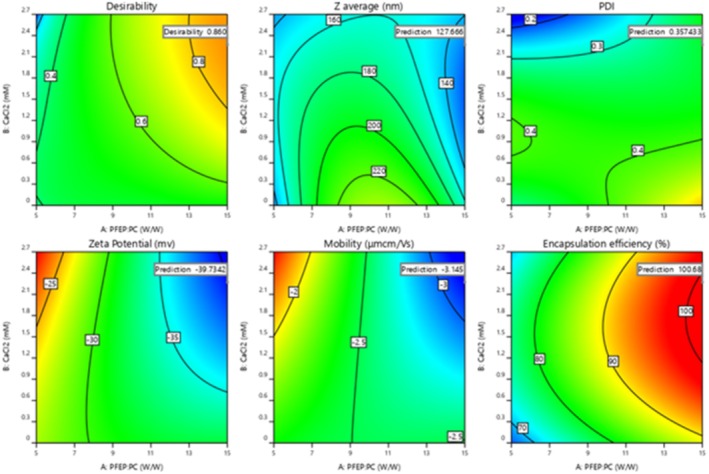
Desirability function response surface for particle size, PDI, ζ‐potential, mobility, and encapsulation efficiency of nanophytosomes loaded with PFE using various PFE: PC ratios, CaCl_2_ and different PFE‐NP: Solvent ratios and their contour plots of the desirability of optimum conditions.

The contour plots provided illustrate the interactions between two crucial factors—Factor A (PFE: PC ratio) and Factor C (PFE‐NP: solvent)—and their effects on multiple responses related to the formulation of nanophytosome, including desirability, *z*‐average size, polydispersity index (PDI), ζ‐potential, mobility, and encapsulation efficiency. General observations indicate that each plot demonstrates how variations in Factors A and C influence these responses, with optimal regions highlighted by contour lines. In the desirability plot, the maximum value of 0.860 indicates highly favorable formulation conditions, where a balance between both factors yields the best outcomes. The *z*‐average size plot predicts an optimal particle size of approximately 127.67 nm, with blue and green areas reflecting smaller sizes, while yellow and red areas show larger sizes, suggesting that optimal conditions are slightly to the left of center. The PDI plot indicates an optimal value of around 0.357, reflecting a narrow size distribution achievable at moderate levels of the factors. The ζ‐potential plot predicts a value of −39.73 mV, indicating adequate stability due to strong electrostatic repulsion among particles, which is maintained within specific concentration ranges. In the mobility plot, the predicted mobility of −3.145 μcm/Vs suggests potential adverse effects on mobility in certain areas, emphasizing the need for careful optimization. Finally, the encapsulation efficiency plot predicts a high efficiency of 100.68%, highlighting that optimal loading of active ingredients is attained at specific concentrations that effectively balance the encapsulating agents and solvent. Overall, these findings underscore the importance of optimizing the interactions between PFE and PFE‐NP: solvent to enhance the stability and efficiency of nanophytosome formulations (Figure 2).

### 3.6 Comparison of Predicted Results With Experimental Outcomes

The optimized treatment was found at a ratio of 1:3 PFE:PC, 2.269 mM CaCl_2_ concentration, and 0.82 PEF‐NP solvent ratio which was obtained by Design‐Expert software for the formulation of nanophytosomes, alongside the corresponding experimental outcomes. The optimal composition yielding a predicted particle size of 127.67 nm, a polydispersity index (PDI) of 0.357, zeta potential of −39.7 mV, mobility of −3.145 which properly explained a well‐distributed and stable formula. Additionally, the expected mobility was −3.145 μmcm/Vs, indicating that electrostatic stability is maintained within the system. The encapsulation efficiency was predicted to be an impressive 100.68%, reflecting optimal loading conditions at this specific formula. The desirability score of 0.860 signifies highly favorable conditions for achieving the desired outcomes. In comparison, the experimental results closely matched these predictions, confirming the robustness of the formula parameters and underscoring the effectiveness of the optimization process in enhancing the stability and performance of the nanophytosomes.

## 4 Conclusion

This study successfully formulated calcium‐reinforced nanophytosomes using bioactive compounds derived from the whole fruit of the Raver Kerman Red Seed cultivar of pomegranate ( *Punica granatum* L.). By optimizing the pomegranate extract to phosphatidylcholine (PC) ratio, calcium chloride concentration, and ethanol solvent ratio through Response Surface Methodology (RSM) and Box–Behnken design in Design Expert software, we identified an optimal formulation with an extract ratio of 1:3 (w/w), 2.70 mM calcium chloride, and a solvent ratio of 0.82 (w/w). The resulting nanophytosomes demonstrated a particle size of 127.67 nm and a zeta potential of −39.7 mV, signaling excellent stability through effective electrostatic repulsion. The low polydispersity index (PDI) of 0.357 indicates a uniform size distribution, reinforcing the formulation's reliability. Remarkably, we achieved an encapsulation efficiency of 100%, demonstrating the model's capability to maximize retention of bioactive compounds. The desirability score of 0.860 highlights highly favorable formulation conditions. Statistical analysis revealed robust *R* ^2^ values from 0.94 to 0.98 for the various responses, showcasing the model's strong predictive accuracy. Adequate precision metrics confirmed a high signal‐to‐noise ratio, affirming the suitability of these models for guiding formulation developments. This research underscores the potential of utilizing the complete pomegranate fruit as a source of bioactive compounds in the production of reinforced nanophytosomes, advancing sustainable practices while enhancing the health benefits associated with pomegranate extracts.

Overall, the results provide strong evidence that using the whole fruit extract of the Raver cultivar in reinforced nanophytosomal systems can be a promising approach for the delivery of natural antioxidants. Beyond demonstrating novelty at the level of raw material and formulation, this study offers a reproducible optimization framework with direct relevance to food, pharmaceutical, and nutraceutical applications.

## Author Contributions

**Ramesh Sedighi:** formal analysis (equal), investigation (equal), methodology (equal), writing – original draft (equal). **Ghadir Rajabzadeh:** resources (equal), visualization (equal), writing – original draft (equal). **Ali Rafe:** conceptualization (equal), formal analysis (equal), project administration (equal), supervision (equal), visualization (equal), writing – original draft (equal), writing – review and editing (equal).

## Conflicts of Interest

The authors declare no conflicts of interest.

## Supporting information


**Data S1:** fsn371229‐sup‐0001‐DataS1.docx.

## Data Availability

Data will be made available on request.
