# Supplementary material for: Optimizations of Calcium‐Reinforced Nanophytosome of Pomegranate Extract Using Box–Behnken Design
Source: Food Sci Nutr. 2025 Nov 25;13(12):e71229. doi: 10.1002/fsn3.71229 (PMC12645159; doi:10.1002/fsn3.71229)
Supplement: Supplementary file 1 — Data S1: fsn371229‐sup‐0001‐DataS1.docx. [file FSN3-13-e71229-s001.docx]

**Supplementary Files**

**Fig.1.** Standard curve for antioxidant activity - DPPH free radical inhibition (IC_50_ = 280.69 mg/L).

**Fig.2.** Standard curve of galic acid in deionized water and ethanol.

**Fig.3.** Standard curve of Flavonoid.
